# The association of microplastics with water-stable aggregates formed under controlled conditions

**DOI:** 10.1039/d6em00213g

**Published:** 2026-09-24

**Authors:** Mike Rohling, Iso Christl, Denise M. Mitrano

**Affiliations:** a Department of Environmental Systems Sciences, ETH Zürich Universitätstrasse 16 8092 Zürich Switzerland denise.mitrano@gmail.com

## Abstract

Water-stable aggregates (WSAs) are a key indicator of soil structural stability and influence water erosion potential. Microplastics (MPs) have been shown to impact WSA formation and their association with WSAs may strongly influence their transport in soils. However, the effect of MPs on WSA formation and their simultaneous association with WSAs has not yet been addressed. This study assessed how MP polymer composition [polylactic acid (PLA) and polyethylene terephthalate (PET)], size (<63 µm and >250 µm) and concentration (0.2, 0.5, and 1 wt%) as well as soil texture (loam and loamy sand) affect WSA formation and MPs–WSA association over a two-week laboratory incubation. The formation of WSAs was decreased most (*i.e.*, 10–13%) by small PLA fragments at low to medium concentrations. Other treatments showed weak or no effects. Across all incubations, MPs were predominantly associated with WSAs (>65%), whereas large PLA fragments at low and small PLA fragments at high spiking concentrations showed comparatively less association. Collectively, MP polymer type, size, and concentration in soil impacted WSA formation and their association with WSAs, implying that the mutual interactions of MP presence and aggregation processes in soils have the potential to affect aggregate stability and particularly MP transport.

## Abstract 2

Microplastics become associated with water stable aggregates, which may impact their transport and fate in soils.

## Graphical abstract

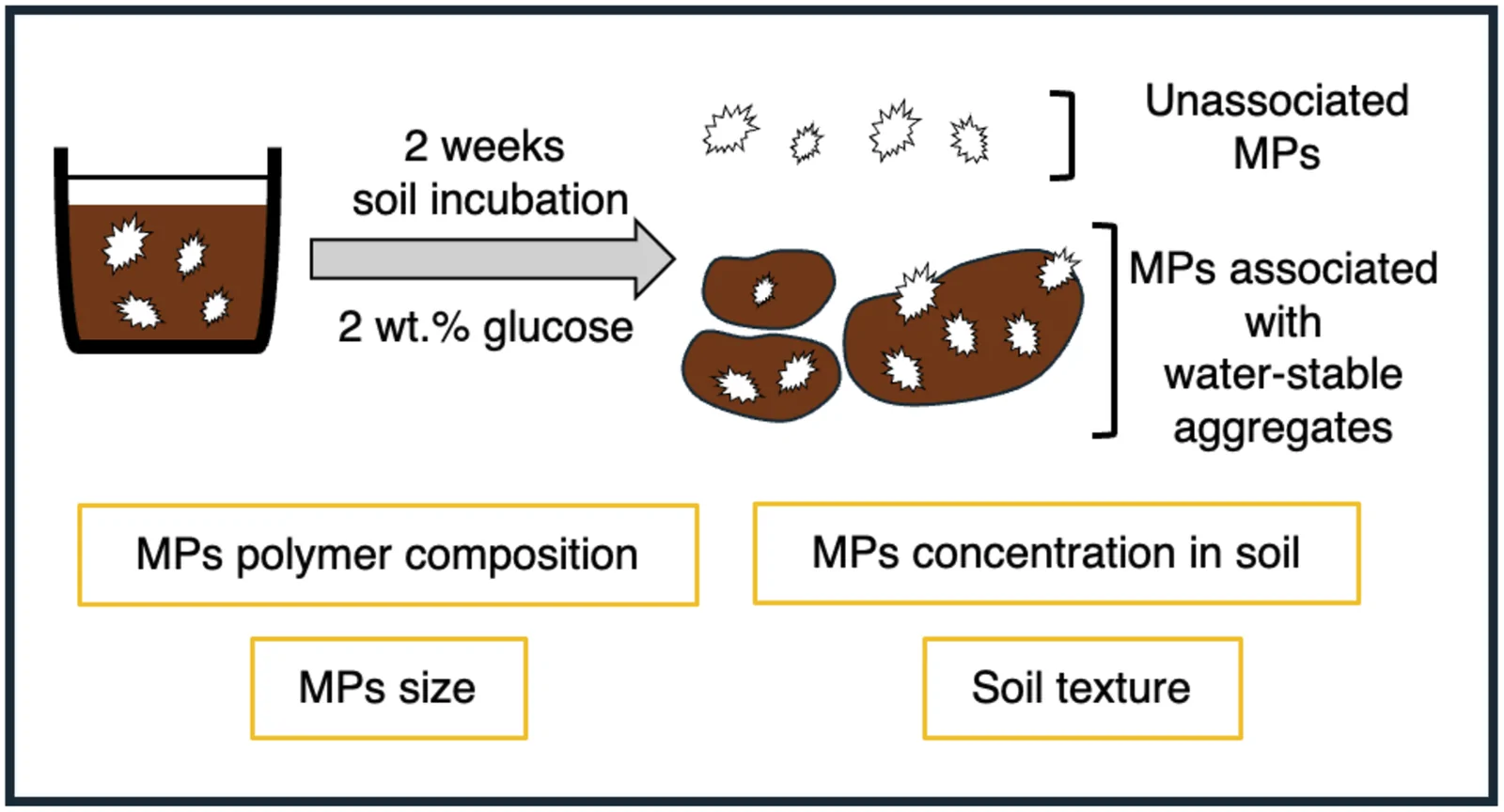

**Environmental significance**

Under natural conditions, most primary soil particles are present in aggregates. Water-stable aggregates (WSAs) are soil aggregates that are able to resist defined water and mechanical stressors that may occur through rainfall or agricultural practices, for example. To date, many studies on MP behavior in soils have not considered that MPs in soils may not be present as isolated particles but rather associated with WSAs. Association of MPs with WSAs has profound implications on the behavior of MPs in soil environments such as *e.g.*, vertical and horizontal transport, bioaccessibility, and biodegradability. This study provides insights into the extent to which MPs become associated with WSAs depending on MP characteristics as well as soil texture.

## 1. Introduction

Soils perform essential functions such as water storage, filtration, and maintaining biodiversity, making their protection and conservation important. Agricultural and industrial activities introduce a variety of stressors to soils, including soil erosion and pollution, both being listed amongst the most serious global soil threats.^1^ Soil loss from cropland induced by water erosion was estimated to be 17 Pg in 2019.^2^ The potential for soil water erosion is closely related to the soil structure, in particular the abundance and size distribution of water-stable aggregates (WSAs). The abundance of water-stable macroaggregates (>250 µm diameter) has therefore often been reported to serve as an indication for the water erosion potential of a soil.

According to the hierarchy model established by Tisdall and Oades (1982), microaggregates (<250 µm) form bottom-up from primary particles and silt-sized aggregates (<20 µm) are held together by persistent binding agents, such as humified organic matter, highly disordered aluminosilicates and oxides.^3^ Macroaggregates then form through the coherence of microaggregates, where the binding agents are primarily temporary (fungal hyphae and plant roots) or transient (microbial and plant-derived polymeric substances). This concept was later extended to account for microaggregate formation within macroaggregates with particulate organic matter (POM) serving as a key nucleus for aggregation.^4^ The disruption of soil aggregates by water can, on the other hand, occur through three different mechanisms: slaking, differential swelling, and physicochemical dispersion. In addition, the kinetic energy of raindrops and flowing water imposes mechanical stressors on soil aggregates.^5,6^ The combination of both water and mechanical stressors is mimicked in laboratory settings by wet-sieving procedures, which involve the immersion of soil aggregates on differently sized meshes at a fixed frequency and time duration.^7^ Aggregates retained on meshes >250 µm are classified as water-stable macroaggregates and typically quantified by mass.

Microplastics (MPs), defined as plastics <5 mm, are found in soils worldwide and, due to their persistence and potential (eco-)toxicological effects, are assessed as environmental pollutants.^8–11^ Polyethylene terephthalate (PET) is amongst the most commonly detected MPs in soils, *e.g.*, introduced by sewage sludge application.^12–16^ The aromatic structure of PET contributes to the desired material properties such as thermal stability and rigidity but was found to hamper biodegradability.^17–20^ In contrast, polylactic acid (PLA) is a polymer certified as biodegradable under industrial composting conditions.^21,22^ In agriculture, PLA is used as a mulch film material, providing a potential pathway for its introduction into soils.^23^ As with other polymers, hydrolysis of the ester bonds is a key initial degradation step, which may be hampered at temperatures commonly found in soils, raising concerns about long-term soil pollution.^21,24,25^

The interplay between MPs and soil aggregation processes can be considered from two perspectives: (1) the impacts of the presence of MPs in soils on the formation of WSAs and (2) the extent of association of MPs with WSAs. Previous studies have reported that MPs impact WSA formation depending on MP polymer composition, shape, and concentration as well as soil properties.^26–30^ On one hand, overall increased hydrophobicity within aggregates was proposed to increase their stability when exposed to water.^28,29^ On the other hand, decreased aggregate stability was attributed to the introduction of additional fracture points by the comparatively inert MP surfaces, as well as reduced microbial activity caused by leaching of plastic-added chemicals.^28,30,31^ These changes in WSA formation indicate that MPs may alter soil water erosion potential by modifying aggregate stability.

MPs have been detected in WSAs in environmental samples, confirming their incorporation under natural conditions.^32,33^ Across five arable soils sampled in southwestern China, 28% of MPs detected were unassociated with WSAs (*i.e.*, free), while the majority of them were associated with WSAs.^33^ The association of MPs with WSAs, particularly macroaggregates, may alter susceptibility of MPs to be water eroded. Rehm *et al.* (2021) investigated MP water erosion under field conditions with large (250–300 µm) and small (53–100 µm) PE fragments and found that fine MPs were transported comparatively less than coarse MPs, which was explained by their stronger association with WSAs.^34^ The association of both small and large MPs with WSAs increased over time (475 days), while the extent of MP erosion simultaneously decreased. Although still underexplored, the association of MPs with WSAs may influence other processes such as the extent of MP physicochemical weathering in soils;^35^ however, the impacts of MPs on WSA formation and their simultaneous association with WSAs have not yet been investigated.

The aim of this study was to investigate the impacts of MP polymer chemistry, size, and concentration in soil on (1) the formation of WSAs to better understand MP-induced alterations in soil structural stability and (2) association of MPs with WSAs, which may impact their transport and fate in soils. To do so, model PET (nominally < 63 µm, hereafter called “small”) and PLA MP fragments (nominally < 63 µm and 250–1000 µm, hereafter called “large”) containing an environmentally rare metal used as a tracer (indium, 0.2 wt%) were spiked into soil which was incubated with an initial 2 wt% glucose treatment to stimulate WSA formation through extracellular polymeric substance (EPS) excretion over two weeks. The use of these model MPs allowed for the measurement of the metal as a proxy for the plastics, easing detection challenges associated with MPs in soil, particularly within complex workflows. Small PET fragments and large PLA fragments were tested at a spiking concentration of 0.2 wt% in a loam-textured soil, while a concentration gradient (0.2, 0.5 and 1 wt%, hereafter called “low”, “medium”, and “high”, respectively) was examined for small PLA fragments. Small PET particles were additionally tested in a coarser textured soil (loamy sand). WSAs formed during the laboratory incubations were isolated by a wet-sieving procedure, quantified by mass and analyzed for MP association. Collectively, the results of this study provide insights into the effects of MPs on WSA formation and MPs–WSA associations and their respective implications, while critically contextualizing these observations within the constraints of laboratory-scale experimentation and environmental relevance.

## 2. Experimental

**Fig. 1:**
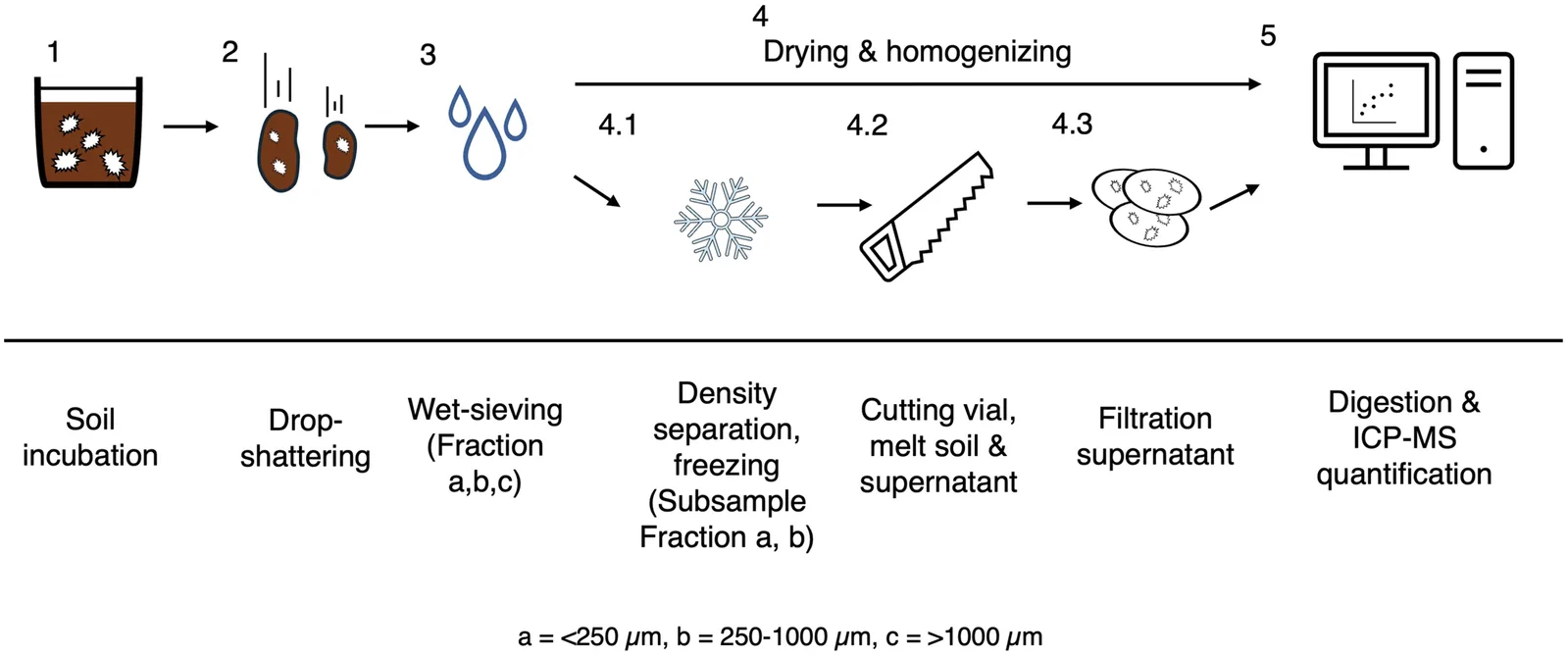
Schematic of the experimental workflow: soils were incubated for two weeks, followed by wet-sieving on 1000 µm and 250 µm meshes. Subsamples of moist fractions <250 µm and 250–1000 µm were taken for density separations in 50 mL tubes. Tubes were deep-frozen, halved, and the supernatant was allowed to melt and then filtered. Remaining soil was oven-dried overnight and homogenized prior to subsampling. Both soil and filter samples underwent microwave-assisted acid digestion and digestates analyzed using ICP-MS.

All soils were incubated for two weeks, drop-shattered until aggregates passed a 10 mm mesh and subsequently wet-sieved on 1000 µm and 250 µm meshes. Moist subsamples of the fractions <250 µm and 250–1000 µm were taken, and a density separation was performed in 50 mL tubes. The tubes were frozen at −80 °C, halved, and the melted supernatants filtered. The remaining soil masses were dried overnight, subsampled, and homogenized. Both the filters and soil samples were then subjected to microwave-assisted acid digestion and analyzed by inductively coupled plasma mass spectrometry (ICP-MS). Details of each step are given below and in schemes of the workflow (Fig. 1 and S1).

### 2.1 Model metal-doped MP fragments

PET and PLA MPs were produced in-house and contained approximately 0.2 wt% In to facilitate their tracing. Further advantages of metal-doped MPs lie in the ease of sample preparation and analysis which are relatively fast compared to other commonly used workflows and quantification techniques.^36^ The exact tracer concentrations are listed in Table S1. Briefly, a metal dopant was compounded into the respective polymer and pelletized, whereafter tracer containing pellets were ground using a cryomill to obtain fragments of different nominal sizes through sequential sieving on 63 and 250 µm mesh sieves. Throughout the manuscript, fragments sieved <63 µm and >250 µm are referred to as “small” and “large”, respectively. Leaching tests conducted with In-doped PET fragments (nominally 63–125 µm) under field conditions showed that >97% of the doped In remained within the material over two years.^9^ Fragments were further characterized by determining the particle size distribution with laser diffraction (Fig. S2) and the specific surface area (SSA) using the Brunauer–Emmett–Teller (BET) method with N_2_ as the adsorbate (see Section S6). As the SSA of large PLA fragments was too small to be measured using the BET method, it was estimated from the particle size distribution under the simplified assumptions of spherical particles (Table S4). Surface acidity was assessed for fine fragments by acid–base titration from pH 3.5 to 7 using HCl and NaOH (Fig. S4). The zeta potential was also determined for small fragments (Table S5).

### 2.2 Soil

The soil used was an alluvial topsoil (0–20 cm) sampled in Emmen (Switzerland) and stored in the dark at room temperature throughout the experiment. The loam soil (USDA texture classification) was prepared by grinding with a pestle and mortar, then sieving to <250 µm. The material retained on the sieve was ground and sieved twice more, with any remaining residue discarded. The sandy loam soil was obtained by following the protocol of De Gryze *et al.* (2006).^37^ Briefly, the ground soil was passed through a 2000 µm mesh stacked on a 250 µm mesh. The fraction 250–2000 µm was mixed with deionized water and shaken overnight on an orbital shaker, then poured onto a 250 µm mesh and rinsed with deionized water. The retained material was treated with 15% H_2_O_2_ (w/v) (Fisher Scientific, Loughborough, UK) and left to react overnight, followed by a combustion treatment at 550 °C to remove residual organic matter. Finally, the fraction 250–2000 µm was recombined with the fraction <250 µm, which had a mass share of 38.3 and 61.7%, respectively. Both soils (loam and loamy sand) were homogenized in a tumbler prior to use. Soil properties are summarized in Table S2.

### 2.3 Soil incubation

All incubations were performed in triplicate in hollow aluminum cylinders (5 cm height and 4.4 cm diameter) covered at the bottom end with aluminum foil held in place with a rubber band. MP spiking and homogenization using a Turbula T2G (Willy A. Bachofen AG, Muttenz, Switzerland) were conducted individually for each replicate. Approximately 25 g of the spiked soil was added per cylinder and gently tapped on a hard surface during packing to ensure uniform packing density. The packing densities were 1.03 g cm^−3^ for the loam and 1.3 g cm^−3^ for the loamy sand, respectively. d-(+)-Glucose (Sigma-Aldrich, pur. > 99.5%, Steinheim, Germany) solution (1 or 2 wt%) or deionized water was then slowly added from the top, using a Pasteur pipette. Due to the different water holding capacities, glucose solutions were concentrated differently for the loam (67 g L^−1^) and the loamy sand (106.4 g L^−1^), respectively. All soils were adjusted to 60% of their gravimetric maximum water-holding capacity. The top end of the cylinder was covered by perforated aluminum foil to allow for gas exchange. Incubations lasted two weeks, with water loss corrected twice (after 7 and 13 days) using deionized water. EPSs were quantified once for the control and the 2 wt% glucose treatment, both in the absence of MPs (see Section S9for details).

### 2.4 Wet-sieving to isolate MPs and WSAs

After incubation, soil columns were removed with a plunger and drop-shattered from 1 m height until all aggregates passed a 10 mm mesh.^38^ The subsequent wet-sieving procedure followed Elliott (1986).^39^ Dry-sieved aggregates were transferred to a 1000 µm mesh and immersed in deionized water for five minutes, followed by manual sieving at 25 immersions per minute for two minutes. Wet-sieving was repeated once on a 250 µm mesh, yielding nominal size fractions of >1000 µm, 250–1000 µm and <250 µm. All fractions were collected in bottles and left undisturbed for 2 days to allow settling of suspended material. Supernatants were then carefully discarded and ∼0.3–6 g aliquots (air-dry mass) were subsampled from the <250 µm and 250–1000 µm fractions to determine unassociated MPs (see Section 2.5). All fraction masses were determined after drying overnight at 80 °C and cooling to constant mass.

To determine the compositions of the 250–1000 µm and >1000 µm fractions of the loamy sand control, soil was passed through 1000 µm (only fraction >1000 µm) and 250 µm sieves by alternating water flushing with manual crumbling of soil. For fraction >1000 µm, the soil passing the 1000 µm sieve was collected and the procedure iterated on a 250 µm mesh. The retained soil was dried at 80 °C overnight to determine the respective mass, which was subsequently subtracted from the total mass of the respective recovered fraction. The mass share of the added mineral fraction 250–2000 µm deviated <1 wt% from the mass share prior to incubation, indicating that the soil <250 µm was successfully removed. The obtained fractions are hereafter called “added fraction 250–1000 µm” and “added fraction >1000 µm”.

### 2.5 Density separation to assess unassociated MPs

Subsamples of moist <250 µm and 250–1000 µm fractions (see Section 2.5) were taken to determine the unassociated fragments. First, they were gently washed into 50 mL tubes with a NaBr (purity >99%, Carl Roth, Karlsruhe, Germany) solution (1.55 g cm^−3^) and made up to 30 mL. Trickling soil into centrifuge vials has been shown to prevent unassociated particles from becoming trapped at the vial bottom.^8^ Our approach followed this procedure but was amended by also gently swirling the vial in a circular motion until all soil was detached from the bottom. The tubes were then centrifuged at 1155*g* for 15 min and deep frozen at −80 °C overnight. Subsequently, the frozen tubes were cut at the 5 mL mark (just above the soil cake) using a handsaw. The top portion containing the supernatant was melted and vacuum filtered through cellulose filters (2–3 µm pore size) to retain any MP fragments. The corresponding soil mass was determined after two washings with deionized water to prevent mass biases from the residual salt. All MPs detected in the supernatant were classified as unassociated. This classification is based on the clearly larger density of the bulk soil (2.47 g cm^−3^), which would cause WSA-associated MPs to settle. After subtracting unassociated fragments in the fraction <250 µm, remaining fragments were classified as associated with WSAs <250 µm.

### 2.6 Sample digestion and analysis for MP quantification

After mass determination, all fractions were ground using a pestle and mortar and subsequently homogenized in a tumbler for 1 min. Subsamples (∼1 g) were subjected to microwave-assisted acid digestion with 7 mL 65–67% HNO_3_ (Sigma Aldrich, Schnelldorf, Germany) using a Turbowave Simultaneous Automated Microwave Digestion System (MWS GmbH, Au, Switzerland). Soil samples were digested in Teflon tubes and filter samples in glass tubes. The digestion protocol for soil followed Schefer *et al.* (2025),^9^ whereas filter digestions were slightly modified to reduced pressure, temperature and time. Indium concentrations in the diluted digestates were determined by inductively coupled plasma mass spectrometry (ICP-MS) using an Agilent 7900 instrument (Basel, Switzerland). Indium standards were purchased from Alfa Aesar (Massachusetts, USA).

### 2.7 Statistical analysis

The recovered soil and MP masses were normalized to 1 (or 100%) for all statistical analyses (see Table S3 for recoveries). Analyses were conducted per fraction, *i.e.*, <250 µm, 250–1000 µm, >1000 µm, and unassociated (only for MP mass). Welch's *t*-tests were performed when two treatments were compared. Data normality was assessed using the Shapiro–Wilk test (*α* = 0.05). For comparisons involving three or more treatments, one-way ANOVAs were executed. Residual distributions and homoscedasticity were assessed using Shapiro–Wilk and Levene's tests (*α* = 0.05), respectively. For all tests, data met the assumptions of normality and homogeneity of variance; therefore, no data transformation was required. Statistical analyses were performed in R 4.5.2 (2025-10-31) using the interface RStudio (2025.05.1+513) using the packages “rstatix” and “car”, in addition to base R.^40^

## 3. Results and discussion

This study required the development of a model system that enabled WSAs to form within relatively short periods under laboratory conditions. WSAs are commonly quantified to describe soil structural stability and serve as a predictor of the water erosion potential of soils. Short-term WSA formation can be promoted by providing labile C-sources, such as glucose, which stimulate the formation of microbial EPSs. The relatively brief incubation under controlled conditions limits potential changes in MP particle properties, such as polymer oxidation and further fragmentation, which may occur over longer time spans. We tested different glucose concentrations on a loam to achieve approximately 50 wt% WSAs larger than the initial sieving size in the control treatment. The glucose concentration that achieved this was likewise applied to a different soil texture (loamy sand) to evaluate the effect of texture on WSA formation. MPs of varying polymer chemistries, sizes, and spiking concentrations were subsequently tested while keeping all other factors constant to determine the effect of each parameter on WSA formation. Finally, MPs were traced to investigate whether, and to what extent, they were associated with different WSA size classes.

### 3.1 Glucose amendments promote WSA formation independent of soil texture

**Fig. 2:**
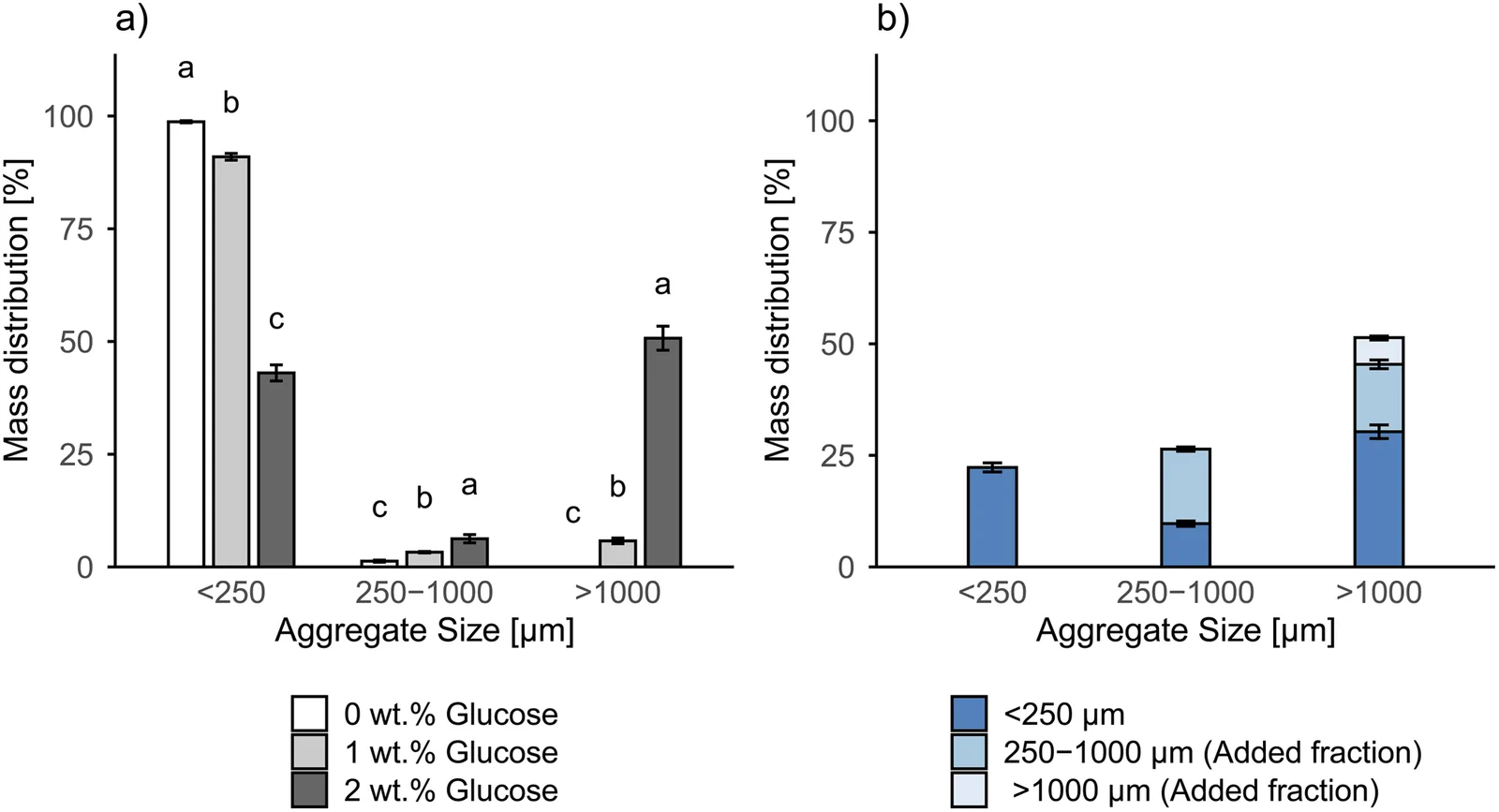
Mass distribution of soil across size fractions (<250 µm, 250–1000 µm, and >1000 µm) depending on glucose concentrations for the (a) loam and the (b) 2 wt% glucose treatment for the loamy sand, including respective shares of the added fractions 250–1000 µm and >1000 µm. All recoveries (96–98.2%) were normalized to 100%. Bars show arithmetic means and error bars represent the standard deviation of experimental triplicates. Distinct letters indicate statistically significant differences (*p* < 0.05) between treatments based on Tukey post-hoc testing.

To test the impacts of glucose amendments on the formation of WSAs, soil was sieved to <250 µm, resulting in a loam soil texture. Aggregates that were retained on sieves after wet-sieving were classified as newly formed WSAs. Control incubations with deionized water resulted in only minimal formation of WSAs exceeding the initial sieving size (Fig. 2a). Over 98% of the soil mass remained in the fraction <250 µm, while WSAs >1000 µm were not formed. In contrast, the application of 1 and 2 wt% glucose at the beginning of the incubation steadily increased WSA formation. Approximately 43% of the recovered soil mass was recovered in fraction <250 µm, meaning that approximately 57% were found in WSAs >250 µm (WSAs 250–1000 µm + WSAs > 1000 µm) after adding 2 wt% glucose. This increase was likely caused by the excretion of extracellular polymeric substances (EPSs) from native soil microorganisms which subsequently promoted WSA formation. This assumption is substantiated by the significantly higher total organic carbon (TOC) concentration in the extracted EPSs of the 2 wt% glucose treatment compared to the control with deionized water (Fig. S5a). Both the application of glucose as a labile C-source that promotes EPS excretion of soil microorganisms and the direct application of EPSs (xanthan gum and alginate) were reported to cause the formation of WSAs.^41^ As suggested in the soil aggregate hierarchy model by Tisdall & Oades (1982), EPSs are considered to be transient binding agents, mainly involved in the formation of water-stable macroaggregates (>250 µm), which is consistent with our findings.^3^

The effect of the 2 wt% glucose amendment was additionally tested on a loamy sand, which was obtained by mixing the mineral fraction 250–2000 µm with the loam. Assessing WSA formation in the loamy sand required a correction for the added coarse fraction, as indicated in Fig. 2b. Soil <250 µm that was present in the fractions 250–1000 µm and >1000 µm signified an incorporation into WSAs, as it otherwise should have passed the 250 µm sieve during wet-sieving. The same holds true for the added fraction 250–1000 µm that was detected in the fraction >1000 µm. Soil <250 µm accounted for approximately 60% of the recovered mass of the loamy sand, while approximately 40% was incorporated in WSAs >250 µm after the incubation. More than half of the 250–1000 µm added fraction was found in the aggregate size >1000 µm, which accounted for 15 wt% of the recovered soil mass. Combining these results shows that at least 55% of mass of the loamy sands was recovered in newly formed WSAs, which was similar to the loam. Both organic matter and clay content are substantially contributing to WSA formation, which clearly showed lower shares in the loamy sand compared to the loam (S2). While comparatively less WSAs have been reported for coarser soil textures under field conditions, several studies could not replicate this finding under short-term laboratory conditions (approximately between two and four weeks).^37,42–45^ De Gryze *et al.* (2006) hypothesized that this was due to the focus on WSA formation through EPSs in laboratory studies, while other, long-term stabilizing mechanisms were not accounted for and might be more texture dependent.^43^ Another factor that may promote WSA formation in coarser textured soils is the facilitated growth of fungal hyphae, which are able to enmesh particles over longer distances.^3,4,44,46^ In conclusion, the addition of 2 wt% glucose resulted in a substantial formation of WSAs during a two-week incubation, independent of soil texture, and was therefore chosen for all MP incubation. While these findings agree well with results from short-term laboratory incubations, soil texture may play a more pronounced role under field conditions.

### 3.2 MP polymer composition affects formation of WSAs

**Fig. 3:**
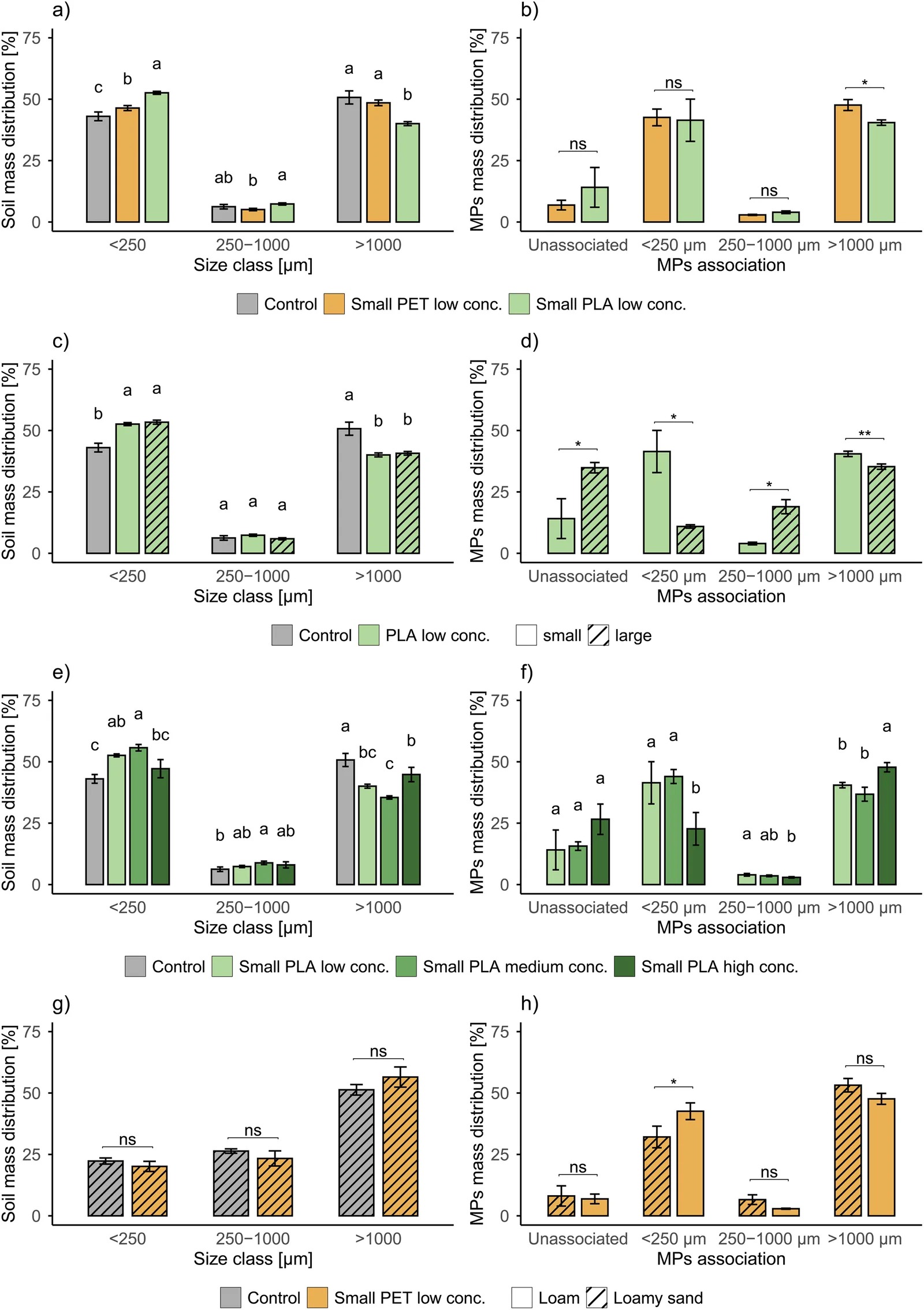
Mass distribution of soil and MPs across fractions (<250 µm, 250–1000 µm, >1000 µm, and unassociated) based on MP polymer chemistry (a and b), MP fragment size (c and d), MP spiking concentration in soil (e and f), and soil texture (g and h). All recoveries (soil: 96–98.2% and MPs: 87.4–102.6%) were normalized to 100%. Bars indicate arithmetic means and error bars the standard deviation of experimental triplicates. Different letters indicate statistically significant differences (*p* < 0.05) between treatments based on Tukey post-hoc testing. For fractions with two treatments, Welch's *t*-tests were performed. Asterisks indicate significant differences (*p* < 0.05 = *; *p* < 0.01 = **). Note that due to analytical constraints, unassociated MPs for large PLA fragments in fraction <250 µm were not determined.

**Fig. 4:**
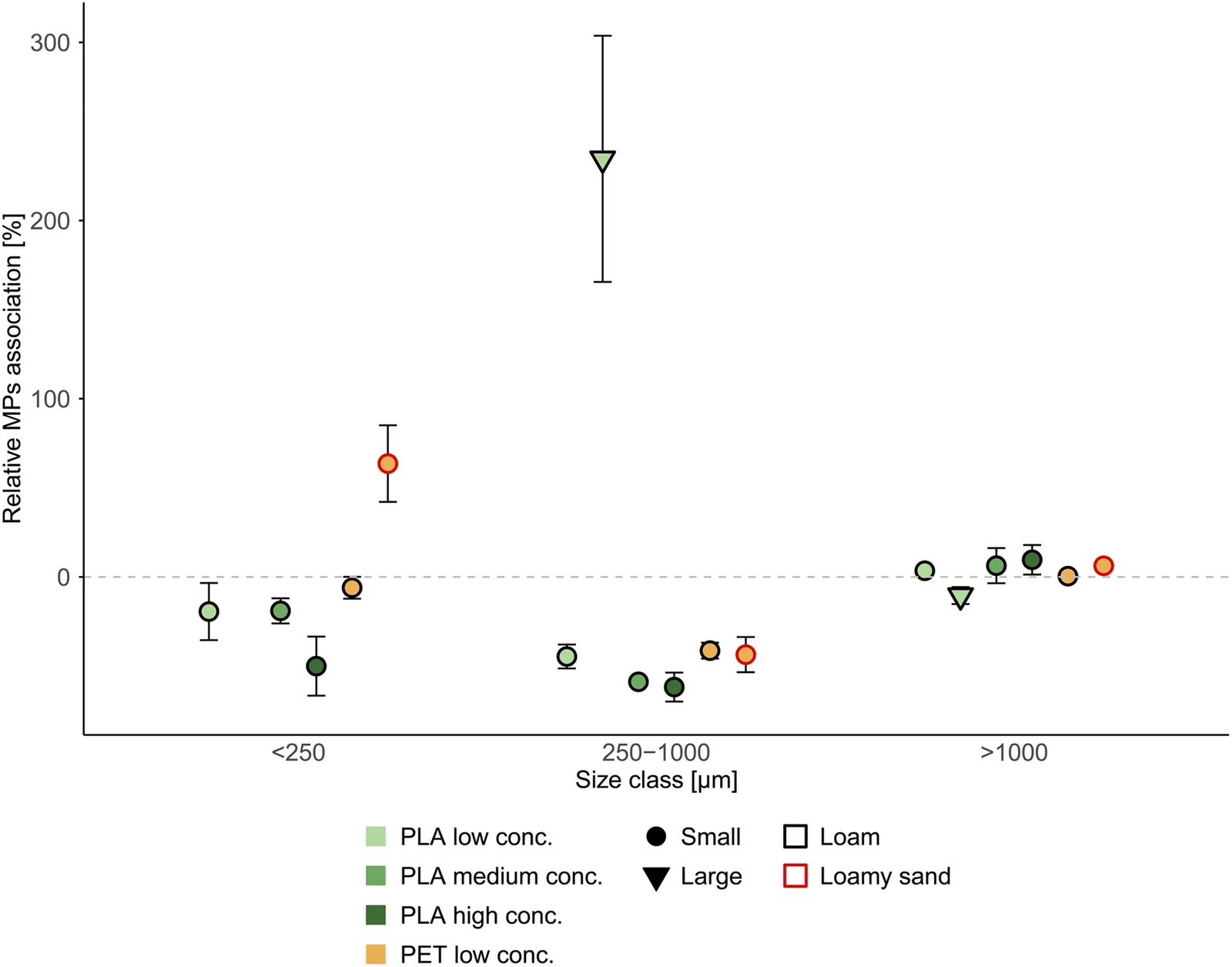
Relative association of MPs in respective aggregate size classes. The dashed line indicates the initial concentration of spiked MPs. Datapoints represent arithmetic means and error bars represent the standard deviation. For the incubation with small PET fragments in the loamy sand, relative association with fractions 250–1000 µm and >1000 µm was corrected for the added fraction 250–2000 µm that may not have been aggregated.

Two different MP polymer compositions, PET and PLA, were investigated at the same nominal size and spiking concentration to assess whether differences in their surface chemistry impacted WSA formation and MP association with WSAs in a loam. Both small PLA and PET fragments reduced WSAs >250 µm formation overall, as indicated by a significantly larger soil share in fraction <250 µm compared to the control (Fig. 3a). Comparatively, the PLA treatment was significantly above the PET treatment in this fraction, indicating that fewer WSAs >250 µm were formed in the presence of PLA fragments. The share of unassociated MPs was roughly twice as high (∼14%) in the PLA treatment, although no statistically significant differences were found (Fig. 3b). Both small PET and PLA fragments showed similar enrichment/depletion in fractions 250–1000 µm and >1000 µm, while PLA fragments were slightly more depleted in fraction <250 µm (Fig. 4).

One possible influence that MPs might have on WSA formation is the release of plastic derived additives that can impair microbial activity. This effect was demonstrated in a soil incubation study using PE and PP films.^31^ However, the additive solution used in that study was obtained by irradiating MPs under UV light in water prior to soil incubation. Since we used pristine MPs and ran incubations for only two weeks, it is unlikely that chemicals, including the doped In_2_O_3_ nanoparticles, were leached from our materials.^9^ Therefore, it is assumed that additive leaching did not impact microbial activity and consequently EPS production under the circumstances investigated here.

Another potential contributing factor to WSA formation is the greater hydrophobicity of MPs compared to soil constituents.^28^ Water enters aggregates through pore spaces, which can be more water repellent in the presence of MPs. The water-contact angle measurement of polymer films showed higher values for PET (84.94 ± 1.64°) than for PLA (79.94 ± 1.3°), meaning PET was slightly more hydrophobic than PLA (S8). It is noteworthy that the hydrophobicity of MPs might have been altered through milling due to changes in surface roughness. Since most mineral surfaces in soils are hydrophilic (*e.g.*, reported water contact angle of quartz surfaces range between 30 and 50°)^47^ and the loam contained only 4 wt% organic matter, the addition of both fragments likely increased the hydrophobic surface area in the soil. This effect alone should have facilitated the formation of WSAs due to hampered water infiltration into the aggregate, suggesting further mechanisms impairing WSA formation.

It has been suggested that MPs act as fracture points in aggregates, thereby reducing particle cohesion, and facilitating their disruption under water- and mechanical stress.^28,30^ Since EPSs may have functioned as the prevailing binding agent under the conditions investigated here, their adsorption behavior onto MP surfaces is likely to influence overall aggregate stability. At soil pH 7, EPSs carried a net negative charge, as indicated by zeta-potential measurements (S7). Small PET and PLA fragments were also slightly negatively charged, confirmed by both their zeta potential and proton buffer capacity, with PLA exhibiting a slightly more negative surface charge than PET. This would imply weaker electrostatic repulsion between EPSs and PET surfaces in comparison to PLA surfaces. However, the loam's negative charge exceeded that of the polymers by approximately four orders of magnitude on a mass base, suggesting that electrostatic repulsion was largely mitigated – likely by cation bridging through polyvalent cations, with Ca^2+^ showing the highest abundance in the investigated soil (Table S6).^48,49,51–53^

Although electrostatic interactions represent a comparatively strong interaction, the hydrophobic effect has often been reported to be the prevailing mechanism driving adsorption of organic (macro)molecules onto MP surfaces.^49,50,54–57^ The more hydrophobic PET surface might conclusively have (1) caused more water repellency and (2) facilitated EPS adsorption through hydrophobic effects and consequently did not affect WSA formation to the extent as was observed for PLA. The results suggest that the surface chemistry of MPs can be an important property when assessing MP impacts on soil structural properties, such as WSA formation.

### 3.3 Large PLA fragments are less associated with WSAs

Soil aggregation processes have previously been reported to be particle size-dependent.^3,58^ As particle size decreases, the surface-area-to-volume ratio increases, providing more adsorption sites per unit volume. To isolate the effect of different MP fragment sizes, large PLA fragments were spiked into the loam at an identical mass fraction (0.2 wt%) and compared with small PLA fragments. The addition of large PLA fragments did not affect WSA formation compared to the small fragments, as no significant differences were detected in any of the size fractions investigated (Fig. 3c). As observed for the small fragments, large ones reduced overall formation of WSAs >250 µm compared to the control. The mass of large MPs associated with WSAs >1000 µm was significantly lower, which was accompanied by a slight depletion in this fraction (Fig. 3d and 4). In contrast, large MPs were significantly more associated with WSAs 250–1000 µm. This fraction was enriched in large MPs by approximately a factor of three, which is assumed to be biased by the comparatively large particle size overlap of the WSA fraction (250–1000 µm) and the MP fragment sizes (Fig. S2) investigated here. The share of unassociated MPs in the large PLA fragment treatment did not only significantly exceed that of small ones but also had the highest share amongst all treatments investigated.

Spiking large rather than small PLA fragments at the same mass concentration altered both the number of introduced particles and the SSA, which was estimated from the particle size distribution under the assumption of spherical particles (Table S4). Adding 0.2 wt% large fragments to soil resulted in one order of magnitude fewer particles per replicate compared to small ones, corresponding to approximately 10^9^ particles. The soil matrix was therefore likely less fragmented by MPs, leaving more extensive contiguous soil regions. The estimated SSA of the large fragments was also one order of magnitude below that of the small ones, compared to both the experimentally determined and calculated SSA (Tables S4 and S6). The combination of less interrupted soil matrix and smaller SSA might have mitigated the soils' hydrophobicity rise through large PLA fragment addition, which potentially contributed to the observed reduction in WSA formation. This is in line with a study where the water-drop penetration time (a measure for a soil’s hydrophobicity) was strongly increased by spiking small MPs (PE, 35 µm) but was not affected when particles were larger (125 µm and 500 µm).^28^ The high proportion of unassociated large fragments further supplements the hypothesis that MPs act as fracture points within soil aggregates. Owing to their lower SSA, larger fragments offer fewer adsorption sites per unit mass, potentially obstructing soil aggregation processes.^58,59^ The extent to which MPs function as fracture points within soil aggregates may consequently be dependent not only on their surface chemistry but also on their size. Comparatively less association of smaller MPs (Polyethylene 53–100 µm *vs.* 250–300 µm) with WSAs was also reported in a water-eroded soil under field conditions.^34^ Summarizing, small and large fragments reduced WSA formation to a similar extent, while the proportion of unassociated fragments was clearly higher when large PLA fragments were spiked into the soil.

### 3.4 Increasing PLA MP concentrations have a non-steady impact on WSA formation

Spiking small PLA fragments at low concentration clearly reduced WSA formation. To better understand the relationship between MP concentration and soil and WSA formation and MPs–WSA association, small PLA fragments were spiked into the loam at medium (0.5 wt%) and high (1 wt%) concentrations. Compared to the low concentration treatment, the medium concentration treatment showed a further reduction in the formation of WSAs >250 µm, as indicated by a larger soil proportion in fraction <250 µm (Fig. 3e). However, there were no significant effects in any of the investigated WSA size fractions. In contrast, the high concentration treatment resulted in the strongest formation of WSAs >250 µm among the tested spiking levels. Although the soil mass share in the <250 µm fraction remained above the control, this difference was not significant.

As observed for the small PLA fragments at low concentration, medium and high concentrations showed neither enrichment nor depletion in WSAs >1000 µm (Fig. 4). There was a slightly stronger depletion in WSAs 250–1000 µm at medium and high concentrations. In WSAs <250 µm, both the 0.2 and 0.5 wt% treatments were depleted by ∼20%, whereas the 1 wt% treatment was depleted by ∼50%. This was also reflected in the highest share of unassociated MPs in the high concentration treatment (Fig. 3f). Thus, this treatment simultaneously exhibited the largest share of unassociated fragments and the strongest formation of WSAs among the tested concentrations.

As discussed above, MPs may affect WSA formation through opposing mechanisms. Their comparatively low charge and polarity may limit the adsorption of binding agents such as EPSs, causing them to act as fracture points within soil aggregates. Conversely, low charge and polarity imply hydrophobicity, which might enhance soil aggregate stability under water-stress conditions. It is therefore hypothesized that the relative contribution of the respective mechanism is dependent on the MP spiking concentration. Following this interpretation, the relative contribution of the hydrophobicity introduced by MPs was the largest at high spiking concentration. This may be supported by a study reporting a strong increase in water-drop penetration time for PE and polybutylene adipate terephthalate (PBAT) fragments (10–300 µm) when MP spiking concentrations were increased from 0.5 to 1 wt% in a disturbed loam.^60^ The simultaneously increasing share of unassociated fragments, nevertheless, points towards declining particle cohesion at the high spiking concentration. Testing PLA fragments along a concentration gradient showed that WSA formation and MP association with WSAs do not follow steady patterns, suggesting the presence of opposing mechanisms through which MPs affect WSA formation.

### 3.5 Small PET fragments do not affect WSA formation in coarse textured soil

Spiking small PET fragments at low concentration had only minor impacts on the formation of WSAs in the loam. To assess whether WSA formation and association of MPs with WSAs were impacted by soil texture, small PET fragments were also spiked into loamy sand at the same concentration. No significant changes in soil mass distribution were observed across the investigated size fractions (Fig. 3g), indicating that the addition of small PET fragments did not alter WSA formation in the loamy sand. Similar to all treatments with small fragments, no MP depletion or enrichment occurred in WSAs >1000 µm while WSAs 250–1000 µm were depleted by approximately 40% (Fig. 4). However, the strongest enrichment in WSAs <250 µm with approximately 60% was observed in this treatment. Despite this strong enrichment, there was a significantly larger share of MPs associated with WSAs <250 µm in the loam compared to the loamy sand (Fig. 3h).

The added mineral fraction 250–2000 µm accounted for approximately 38 wt% of the loamy sand, leaving 62 wt% as soil <250 µm. Since the PET fragments were <63 µm, they may be concentrated in this fraction when classified by size, such as by wet sieving. Adjusted to the soil fraction <250 µm, the effective spiking concentration was increased by roughly 60% compared to the bulk concentration, which may explain the 60% enrichment observed in WSAs <250 µm. Due to their comparatively low SSA, sand grains are not assumed to actively contribute to soil aggregation, underscoring the importance of the <250 µm fraction.^45^ Despite the comparatively higher MP concentrations in this fraction, WSA formation was not altered compared to the control, while the share of unassociated MPs resembled that of the loam incubation. However, less MP association with WSAs in coarser soil textures has been reported under field conditions, implying that laboratory studies might not capture all environmentally relevant factors.^34^

### 3.6 Linking laboratory experiments to environmental impacts

The results of the experiments performed here showed that both WSA formation and the association of MPs with WSAs are influenced by MP properties and concentration in soil. However, it is crucial to contextualize these experiments to evaluate their applicability to natural systems. By systematically varying the polymer chemistry, particle size, spiking concentration and soil texture, while keeping other factors constant, this study established a mechanistic framework for evaluating how these key MP characteristics shape WSA formation and MPs–WSA association. However, several properties of this system were manipulated to ensure controllability and practical feasibility. As reviewed by Abiven *et al.* (2009), monosaccharide soil amendments, such as glucose, proved to be most efficient in promoting substantial WSA formation within short time spans.^61^ While glucose represents the most common sugar in soils, glucose concentrations comparable to those used in this experiment are unlikely to occur in natural systems.^62^ Nevertheless, the majority of the added glucose was likely mineralized to CO_2_ or incorporated into biomass by the time the incubation was finished: after two weeks, the glucose equivalents in the 0.01 M CaCl_2_ extract were approximately two orders of magnitude below the initially added glucose concentration of 20 mg g^−1^ (S9). Although there was a significant increase of ∼1 mg C per g soil in the EPS fraction (0.1 M EDTA extract, dialyzed >14 kDa), this would correspond to only approximately 13% of the added glucose C.

Under field conditions, microbial excretion of EPSs remains a crucial step for WSA formation, but is typically stimulated by the introduction of POM, such as plant residues. Easily available C from POM is utilized by microorganisms, causing the production of EPSs and the formation of water-stable macroaggregates (>250 µm) around the POM.^4,63,64^ Upon degradation, the introduced POM further fragments and is successively more present in the microaggregate (<250 µm) fraction. Hence, WSAs are not a static soil property but steadily involved in turnover processes, implying that MPs are likewise dynamically (un)associated with WSAs. Due to the limited biodegradability and the very low surface charge of MPs it is, nevertheless, unlikely that MPs function as aggregation nuclei such as POM.

Due to weathering, MPs present in soils are likely to be physiochemically altered compared to the pristine model materials used for this study.^65^ For instance, UV-weathering can promote the formation of oxygen-containing functional groups, resulting in reduced surface hydrophobicity.^54,66^ Advanced polymer oxidation has also been reported for MPs sampled from soils.^65^ In addition, MP surfaces can be masked by adsorption processes, such as the proposed EPS adsorption in this study. Adsorption of organic macromolecules onto pristine plastics has been shown to substantially reduce surface hydrophobicity.^54^ While the extent of surface masking by adsorption was not assessed in this study, the remaining surface was likely less weathered compared to MPs found in natural environments. It was hypothesized that the introduction of MPs could promote WSA formation through increased water repellence. Due to weathering and partial surface masking through adsorption, this effect may be mitigated under more natural conditions. However, weathering and adsorption modify but do not necessarily override inherent polymer hydrophobicity. For example, UV-weathered polyethylene and polypropylene surfaces were more hydrophobic than pristine PET, even after adsorption of organic macromolecules.^54^ Consequently, MP polymer composition is an important factor to consider when evaluating their effects on WSA formation, also under natural conditions.

While reported MP concentrations in sewage sludge, roadside and industrial soils are comparable to the concentrations spiked in this experiment, concentrations in arable soils were reported to be below the standard spiking concentration (0.2 wt%) used here. While using elevated concentrations is legitimate for gaining mechanistic understanding or overcoming analytical constraints, conclusions for the environment should be drawn with caution. Although some treatments showed statistically significant reductions in formation of WSAs >250 µm, even the strongest observed reduction was only approximately 13% compared to the control (small PLA fragments, low conc., loam). Based on these results, it is reasonable to expect that WSA formation at approximately three orders of magnitude lower MP concentrations would resemble the control level. Consequently, we may not anticipate alterations in WSA formation under current MP burdens in most soil systems. Despite the comparatively high spiking concentrations, MP association with WSAs was >65% across all incubations and tended to increase with lower concentrations, as was observed for small PLA fragments in the loam. It is therefore hypothesized that most MPs of the investigated sizes are likely to associate with WSAs and that the transport of MPs in soil systems may predominantly occur in association with WSAs. While several aspects of aggregate formation, MP properties and concentrations deviated from natural soil conditions, these constraints were deliberately introduced to ensure analytical feasibility and to isolate the key mechanisms governing WSA formation and MPs–WSA association.

## 4. Conclusions

This study examined how the MP polymer chemistry, particle size, and spiking concentration as well as soil texture affected the formation of WSAs >250 µm and to which extent MPs were associated with WSAs. Consistent with earlier studies, MPs can alter WSA formation depending on both MP and soil properties as well as MP concentration.^26–28,30,67^ The observed effects along a concentration gradient followed a non-steady pattern and suggested that MPs impact WSA formation through opposing mechanisms, which need further validation in future studies. However, studies working closer to environmentally relevant concentrations are lacking so far. Current knowledge is therefore primarily tailored for mechanistic understanding or for future scenarios under the assumption of ongoing plastic pollution and degradation of macroplastics to MPs.^68^ Since WSA formation is shaped by the complex interplay of MPs and soil properties, accurate predictions remain challenging. In addition, other factors such as farming practices may play an overall stronger role in determining soil structural stability, including effects on soil water erosion potential.^69^

Across all incubations, most MPs were associated with WSAs, which could strongly impact their transport behavior in soils. When associated with WSAs, MP properties can be masked. The overall density and size of a WSA can substantially exceed that of the associated MPs, likely reducing not only their horizontal transport *via* water erosion, but also their vertical transport. Similar to soil organic matter, occlusion within WSAs may impede MP degradation due to reduced accessibility for microorganisms as well as limited diffusion of enzymes and oxygen.^70^ Shielding from UV-radiation may further limit the continued weathering of MPs. Moreover, occluded MPs are potentially less accessible for the soil fauna, such as nematodes and earthworms. The concurrent reduced mobility and exposure to weathering factors could overall signify that soils are long-term sinks for MPs. To date, many studies on MPs in soils have neglected the potential for association with WSAs and environmental sampling may likewise underestimate if MPs associated with WSAs are excluded.^15^ This study showed that MPs in soil systems are likely to be associated with WSAs, which may critically impact their transport and fate and should therefore receive greater consideration in future studies.

## Author contributions

All authors were involved in the experimental design. MR executed the experimental work and conducted data analyses and visualization. All authors evaluated, discussed and interpreted the data. MR wrote the manuscript, with input from DMM and IC. All authors approved the final version of the manuscript.

## Conflicts of interest

There are no conflicts to declare.

## Supplementary Material

EM-028-D6EM00213G-s001

## Data Availability

The datasets used in this study are published on Zenodo: (https://doi.org/10.5281/zenodo.21391931). Supplementary information (SI): MPs tracer concentration (Table S1); general soil properties (Table S2); MPs and soil recoveries (Table S3); a detailed workflow (Fig. S1); MPs size distribution (Fig. S2 and Table S4); MPs SSA (Fig. S3); MPs surface chemistry (Fig. S4 and Table S5); quantification and charge determination of extracellular polymeric substances (Fig. S5); soil cation exchange capacity (Table S6). See DOI: https://doi.org/10.1039/d6em00213g.
