# Supplementary material for: The association of microplastics with water-stable aggregates formed under controlled conditions
Source: Environ Sci Process Impacts. 2026 Sep 24;28(10):2612–24. doi: 10.1039/d6em00213g (PMC13612103; doi:10.1039/d6em00213g)
Supplement: Supplementary file 1 [file EM-028-D6EM00213G-s001.pdf]

Supplementary Information (SI) for Environmental Science: Processes & Impacts.
This journal is © The Royal Society of Chemistry

Supporting information (SI)

The association of microplastics with water-stable aggregates formed under controlled conditions

Mike Rohling[1], Iso Christl[1], Denise M. Mitrano[1]

[1] Department of Environmental Systems Sciences, ETH Zürich, Universitätstrasse 16, 8092 Zürich, Switzerland

Corresponding author: denise.mitrano@rd.nestle.com

Summary:

Thirteen pages

Five figures (S1–S5)

Six tables (S1–S6)

S1. Microplastics tracer concentration

Table S1: Indium-content of model MPs. MPs were quantified in filter and soil samples after undergoing digestion under conditions identical to those used for the experimental samples.

MPs	Tracer concentration in filter samples (mg In g⁻¹)	Tracer concentration in loam samples (mg In g⁻¹)	Tracer concentration in loamy sand samples (mg In g⁻¹)
PET <63 μm	2.19 ± 0.08	1.8 ± 0.18	1.9 ± 0.1
PLA <63 μm	2.19 ± 0.05	1.97 ± 0.07	/
PLA >250	2.01 ± 0.07	2.12 ± 0.17	/

S2. Soil properties

Table S2: Soil properties of the loam and the loamy sand.

Soil	Sand (wt. %)	Silt (wt. %)	Clay (wt. %)	Organic matter (wt. %)	pH (H_2O)	Maximum water-holding capacity (wt.%)	In-concentration (ng g^{-1})
Loam	36.1	40.2	23.7	3.8	7.4	55	36.13 ± 3.26

Loamy sand	64.8	21.5	13.5	2.5	7.5	35	41.46 ± 4.1

S3. Microplastics and soil recoveries

Table S3: Overview of all incubations with respective microplastics (MPs) and soil recoveries.

Soil texture	Glucose addition (wt.%)	Polymer chemistry	Nominal MPs fragment size (μm)	MPs spiking concentration (%)	MPs recovery (%)	Soil recovery (%)
Loam	2	PET	<63 μm	0.2	95.8 ± 1.6	97.6 ± 0.2
Loam	2	PLA	<63 μm	0.2	86.8 ± 1.7	97.7 ± 0.6
Loam	2	PLA	<63 μm	0.5	94.3 ± 3.7	97.6 ± 0.2
Loam	2	PLA	<63 μm	1.0	89.2 ± 6.2	97.5 ± 0.3
Loam	2	PLA	250–500 μm	0.2	100.6 ± 0.9	96.9 ± 0.7
Loamy sand	2	PET	<63 μm	0.2	102.6 ± 3.8	98.2 ± 0.2
Loam	0	/	/	/		96 ± 0.6
Loam	1	/	/	/		96.3 ± 1.0
Loam	2	/	/	/		96.1± 0.7
Loamy sand	2	/	/	/		98.2 ± 0.5
					94.4 ± 6.2 (Arithmetic mean)	97.2 ± 0.8 (Arithmetic mean)

S4. Detailed experimental workflow

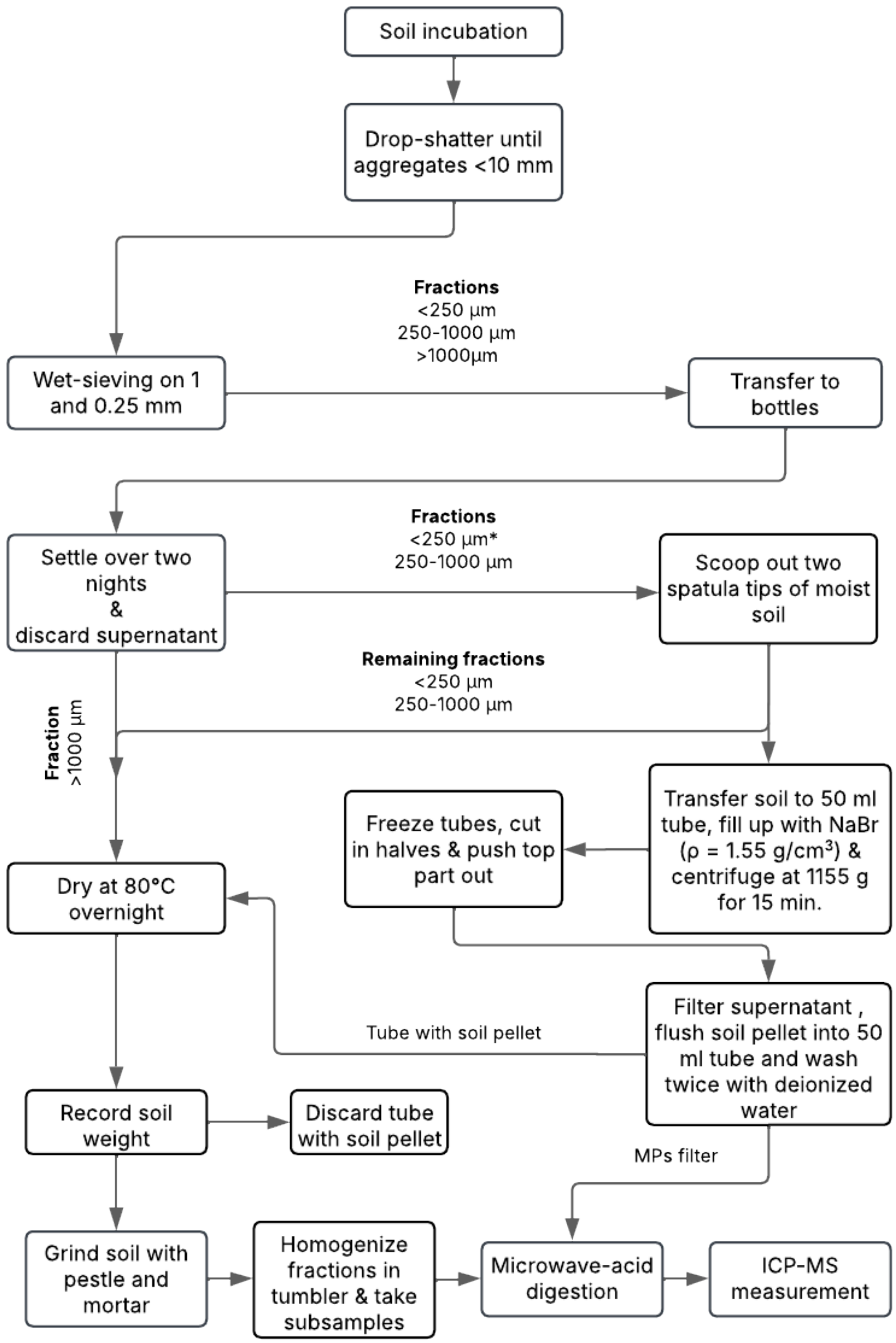

Figure S1: Detailed scheme of the workflow conducted across all experimental replicates. * Indicates that this step was not conducted for the incubation of PLA >250 μm.

S5. Microplastics size distribution

The size distribution of the MPs fragments was determined by laser diffraction, using a LS 13 320 (Beckmann Coulter, Indiana, United States) with a Universal Liquid Module (optical model: Fraunhofer.rf780d at 780 nm wavelength, fluid: ethanol, measuring range: 0.4–2000 μm). Notably, Fraunhofer diffraction assumes spherical particles. Each measurement was conducted with a spatula tip of the respective material.

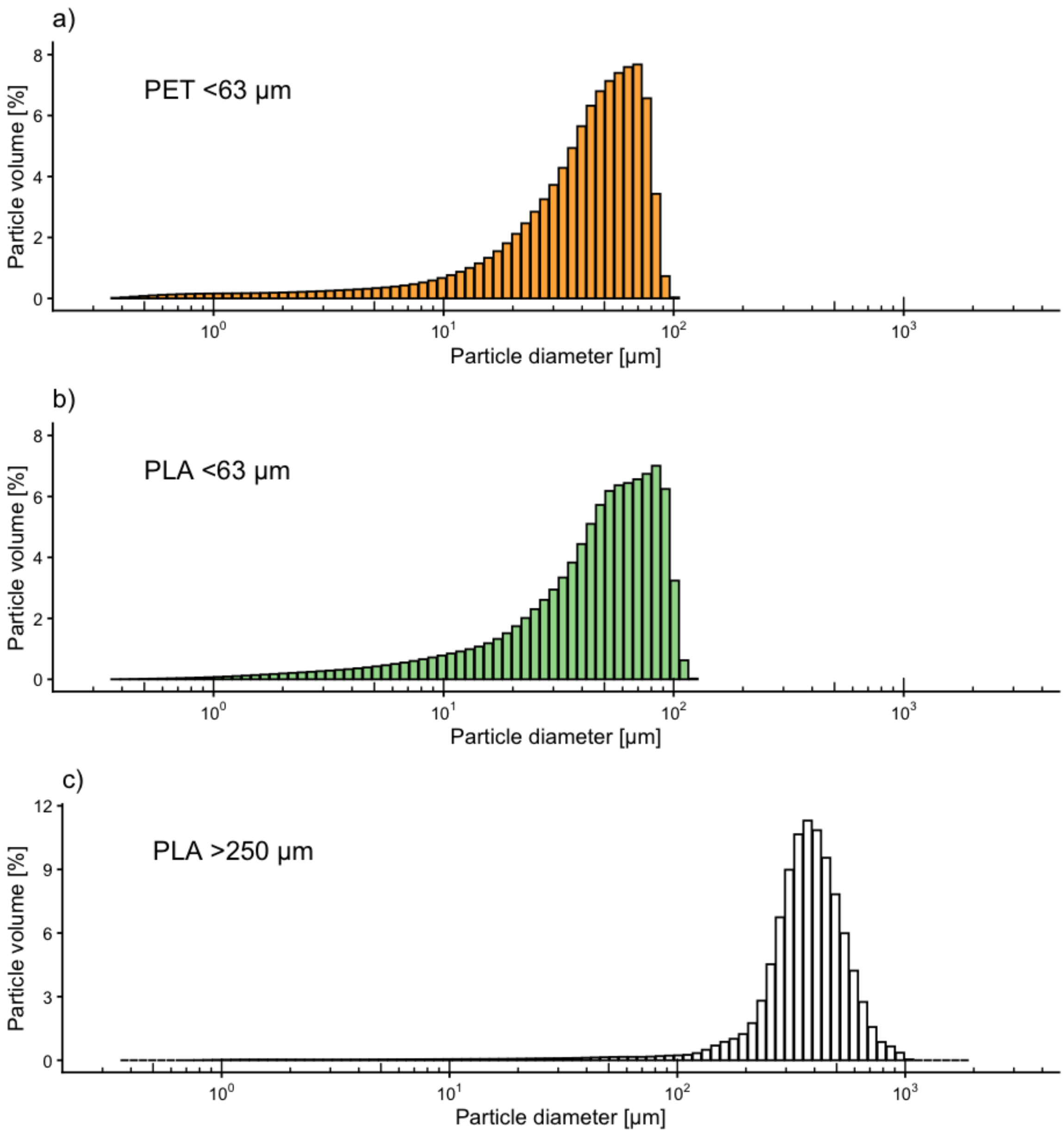

Figure S2: MPs fragment size distribution of (a) PET <63 μm. (b) PLA <63 μm and (c) PLA >250 μm. Particle volume percent is equivalent to the mass percent within the same polymer chemistry.

Table S4: Information on median and mean MPs fragment sizes used for incubation experiments as derived from particle size distribution measurements (Fig. S2). Corresponding fragment numbers and specific surface areas were calculated per gram MPs assuming spherical shapes.

MPs	Median diameter (μm)	Arithmetic mean diameter (μm)	MPs fragment number g^{-1}	Specific surface area ($m^2\ g^{-1}$)
PET <63 μm	46.2	45.7 ± 23.5	$7.51*10^{10}$	0.277
PLA <63 μm	52.2	52.6 ± 228.7	$2.39*10^{10}$	0.233
PLA >250 μm	392.4	403.9 ± 158.2	$2.3*10^{9}$	0.033

S6. Microplastics specific surface area

The specific surface area of PLA and PET fragments <63 μm were determined using N_2 gas adsorption measurements at 77 K with an Autosorb 6100 FKM (Anton Paar, Baden, Switzerland). Prior to measurement, each sample was evacuated at 328 K for 15 h. The specific surface area was evaluated and calculated using the 11-point BET method in the relative pressure range p/p_0 = 0.05–0.3.[1] The specific surface area for PET and PLA fragments was 0.67 $m^2\ g^{-1}$ and 0.542 $m^2\ g^{-1}$, respectively.

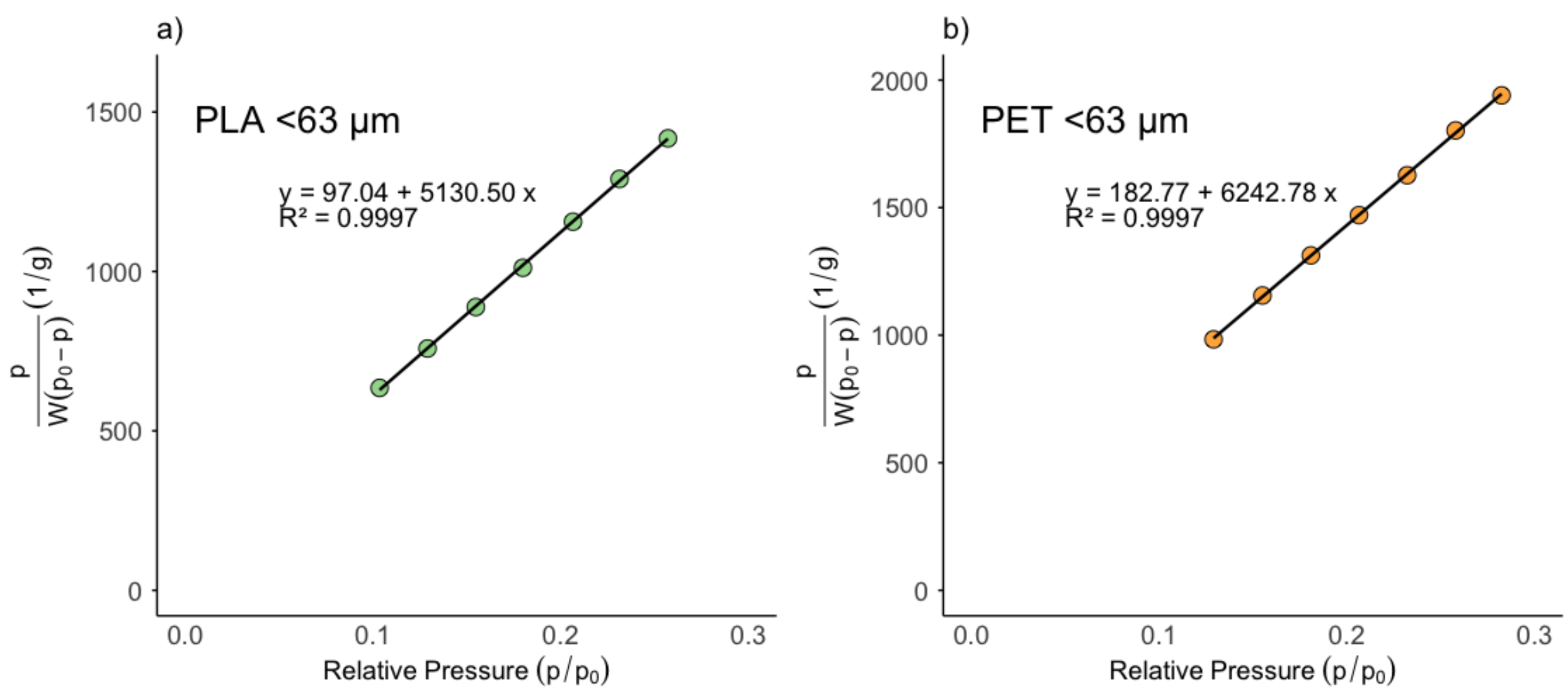

Figure S3: N_2 adsorption onto MPs surfaces of (a) PLA <63 μm and (b) PET <63 μm as a function of the relative pressure, with p=N_2 pressure, po= N_2 saturation pressure, W = adsorbed gas volume and g=sample mass.

S7. Microplastics surface chemistry

The surface chemistry of PLA <63 μm and PET <63 μm fragments was investigated using acid–base titration experiments and zeta potential measurements. Acid–base titration experiments of fragment suspensions were carried out in a NaCl background electrolyte solution using an automated titration setup in a thermostated room at 25 ±1 °C. 0.5 M HCl and 0.5 M NaOH prepared from Titrisol® solutions (Merck, Darmstadt, Germany) diluted with CO_2-free deionized water (Puranity PU 15, VWR International, Leuven, Belgium; 17.4 MΩ*cm) were used as the titrant solutions.[2] The ionic strength was (re-)adjusted to the target value (0.01 M, 0.03 M, and 0.10 M), for different experimental variants after each titrant addition by aliquots of either CO_2-free, deionized water or 1 M NaCl (NORMAPUR, VWR International, Leuven, Belgium) solutions. The headspace of the titration vessel was continuously purged with water-saturated, CO_2-free N_2 gas. The pH values were measured using a pH-glass electrode (6.0123.100, Metrohm, Herisau, Switzerland) and an Ag/AgCl reference electrode (6.0733.100, Metrohm, Herisau, Switzerland) placed in an electrolyte bridge (0.1 M NaCl). The pH electrode readings were recorded once the electrode drift was below 0.03 mV per minute or after a maximum drift time of 20 minutes. The number of protons consumed by the MPs surfaces was calculated for each data point as the difference between total amount of acid and base added and the consumption of protons and hydroxyl ions by the aqueous background solution, as calculated with the speciation program ECOSAT.[3]

At the end of the titration experiments, aliquots of the suspensions were taken for zeta potential measurements using a ZetaPALS (Brookhaven Instruments Corp., Holtsville, NY, USA) zeta potential analyzer. Prior to the measurements, the pH value was adjusted to 3.5 or 7.0 by addition of 0.05 M HCl and 0.05 M NaOH, respectively. A standard suspension, freshly prepared using BI-ZR3 dry powder as recommended by the manufacturer, was used as the reference material yielding a value of −53 ±4 mV in a 0.1 mM KCl background electrolyte.

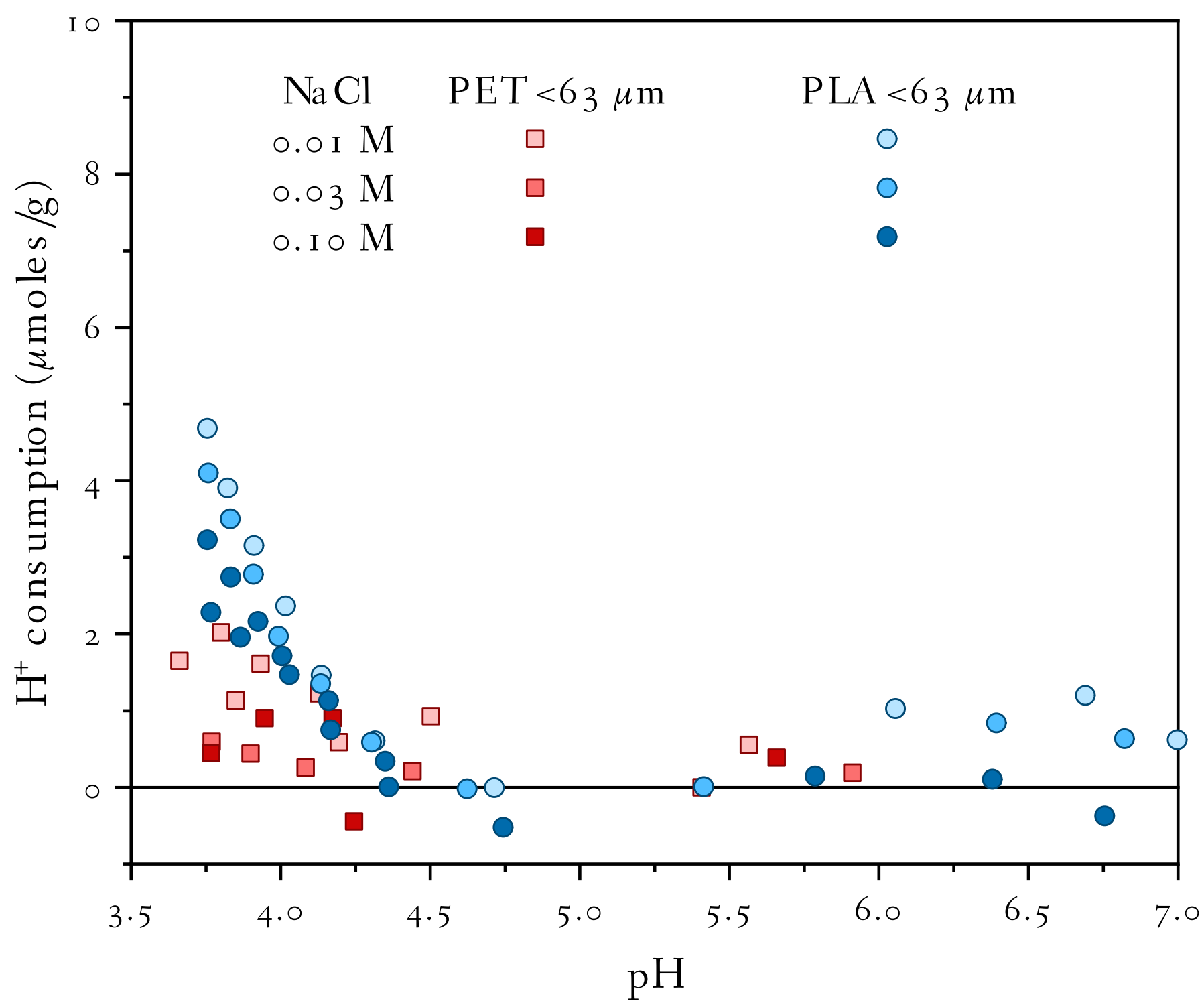

Figure S4: Proton consumption by PET (<63 μm) and PLA (>63 μm) MPs in NaCl background electrolytes, as calculated from acid–base titration data.

The acid–base titration data (Fig. S5) indicated the presence of surface groups that were protonated at pH below 4.5. Considering pK_a values of polymer chain ends corresponding to the respective values of the monomers terephthalic acid (pK_{a1} = 3.54) and lactic acid (pK_a = 3.86), twice the amount of protons consumed at that pH may correspond to the total amount of proton-reactive surface groups of the particles, i.e., about 5–8 μmol/g for PLA (<63 μm) and about 2–3 μmol/g for PET (<63 μm).

The zeta potentials measured in 0.1 M NaCl indicated that both PLA (<63 μm) and PET (<63 μm) MPs were increasingly negatively charged with increasing pH (from 3.5 to 7, Table S2) due to increasing deprotonation of proton-reactive surfaces groups as indicated by the titration data (Fig. S4). Based on acid–base titration and zeta potential measurements, it can be concluded that both MPs chemistries were fully deprotonated at pH >5, with total negative particle charges corresponding to the estimated respective total amounts of proton-reactive surface groups given above.

Table S5: Zeta potentials of PET (<63 μm) and PLA (<63 μm) MPs in 0.1 M NaCl.

pH	ζ (PET <63 μm)	ζ (PLA <63 μm)
3.5	−11.97 ±0.66 mV	−25.43 ±1.25 mV
7.0	−19.88 ±1.96 mV	−34.93 ±0.77 mV

S8. Polymer film hydrophobicity

Surface hydrophobicity was assessed using films prepared from the same original materials as the MPs. The hydrophobicity of both PLA and PET films was determined by measuring the water contact angle with a drop-shape analyzer (DSA30S, Krüss, Hamburg, Germany). Droplets (n=5) of two μl deionized water were deposited onto the film through a syringe and recorded with a device-intern camera. Droplet geometry was measured at the three-phase contact line using an elliptic shape recognition mode (software: Advance, Krüss, Hamburg, Germany) by averaging measured left and right angle. The obtained water contact angeles were 79.94 ± 1.3 for the PLA film and 84.94 ± 1.64 for the PET film, respectively.

S9. Quantification and charge determination of extracellular polymeric substances

The extraction of extracellular polymeric substances (EPS) followed the protocol presented in Bérard et al. (2020) with slight adjustments.[4] Analyses were conducted with the loam after a two-week incubation with either deionized water or 2 wt.% glucose in the absence of microplastics. After the incubation, soils were removed from the cylinder, evenly spread in an aluminum pan, and dried in a fume hood until a constant mass was achieved. Soils were then ground using a pestle and a mortar and homogenized in a tumbler. Five technical replicates of approximately 0.5 g were transferred into 50 mL tubes and filled up with 0.01 M $CaCl_2$ solution at a 1:10 (w/v) ratio, shaken on an overhead shaker for 1 h at room temperature and subsequently centrifuged at 3500 *g* for 30 min. Supernatants were collected, filtered with 0.45 μm membrane filters (add material (Nylon?) and supplier information) and stored at -20°C. The tubes were re-filled with 25 mL 0.1 M Na_2-EDTA and shaken for 16 h on an overhead shaker. Thereafter, tubes were centrifuged at 3500 *g* for 30 min. once more. The obtained supernatants were filtered through 0.45 μm polyethersulfone filters (Whatman, Maidstone, England) dialyzed (Cellulose, 14 kDa cutoff; Sigma-Aldrich, Schnelldorf, Germany) against 0.1 M NaCl for 24 h and subsequently against ultrapure water for 48h under gentle agitation. Dialyzed samples were likewise stored at -20°C.

The zeta potentials of the dialyzed EDTA extracts were measured by dynamic light scattering using a Zetasizer Nano Series (Malvern Instruments Ltd., Malvern, UK). Total organic carbon (TOC) of both $CaCl_2$ and dialyzed EDTA extracts were determined with a Total organic carbon analyzer (Shimadzu, TOC-L, Kyoto, Japan). Protein concentrations were determined in both extracts using the adapted Lowry assay which corrects for absorbance of humic-like substances.[5] Measurements were performed at 750 nm wavelength and calibrated with bovine serum albumin (BSA). A detailed protocol can be found in Redmile-Gordon et al. (2013).[6] (Poly)saccharide concentrations were measured with the DuBois assay.[7] Aliquots of sample solutions (0.5 mL), 5% (w/v) phenol (0.75 mL) and 95.0-98.0% sulfuric acid (2.5mL) were mixed, vortexed and allowed to react for 10 min. Measurements were calibrated with glucose and conducted at 490 nm wavelength. Colorimetric measurements were conducted with an Infinite 200Pro microplate reader (Tecan, Männedorf, Switzerland).

Independent of glucose addition, EPS exhibited a slight negative zeta-potential (control: -19.2 ±2.8 mV, glucose treatment: -17.9 ±3.4 mV), likely due to the presence of negatively charged functional groups such as carboxyl, sulfate, or phosphate. The addition of 2 wt.% glucose at the start of incubation led to a significant increase in total organic carbon (TOC) in the EPS fraction (>14 kDa, 0.1 M EDTA extract; Figure S5 a), corresponding to ∼1 mg g^{-1} soil or 12.5% of the added glucose-C. The non- or loosely bound C pool (0.01 M $CaCl_2$ extract) also increased, though absolute differences were roughly an order of magnitude lower than those in the EPS fraction. These observations suggest that, under excess C supply, soil microorganisms produce high-molecular-weight compounds that may contribute to the formation of water-stable aggregates (Figure 2 in the main manuscript). Protein and polysaccharide concentrations in the EPS fraction also increased, but less than the TOC, showing that the a substantial share of excreted EPS do not fall in the categories of proteins and polysaccharides as detected by the Lowry and Dubois assays.

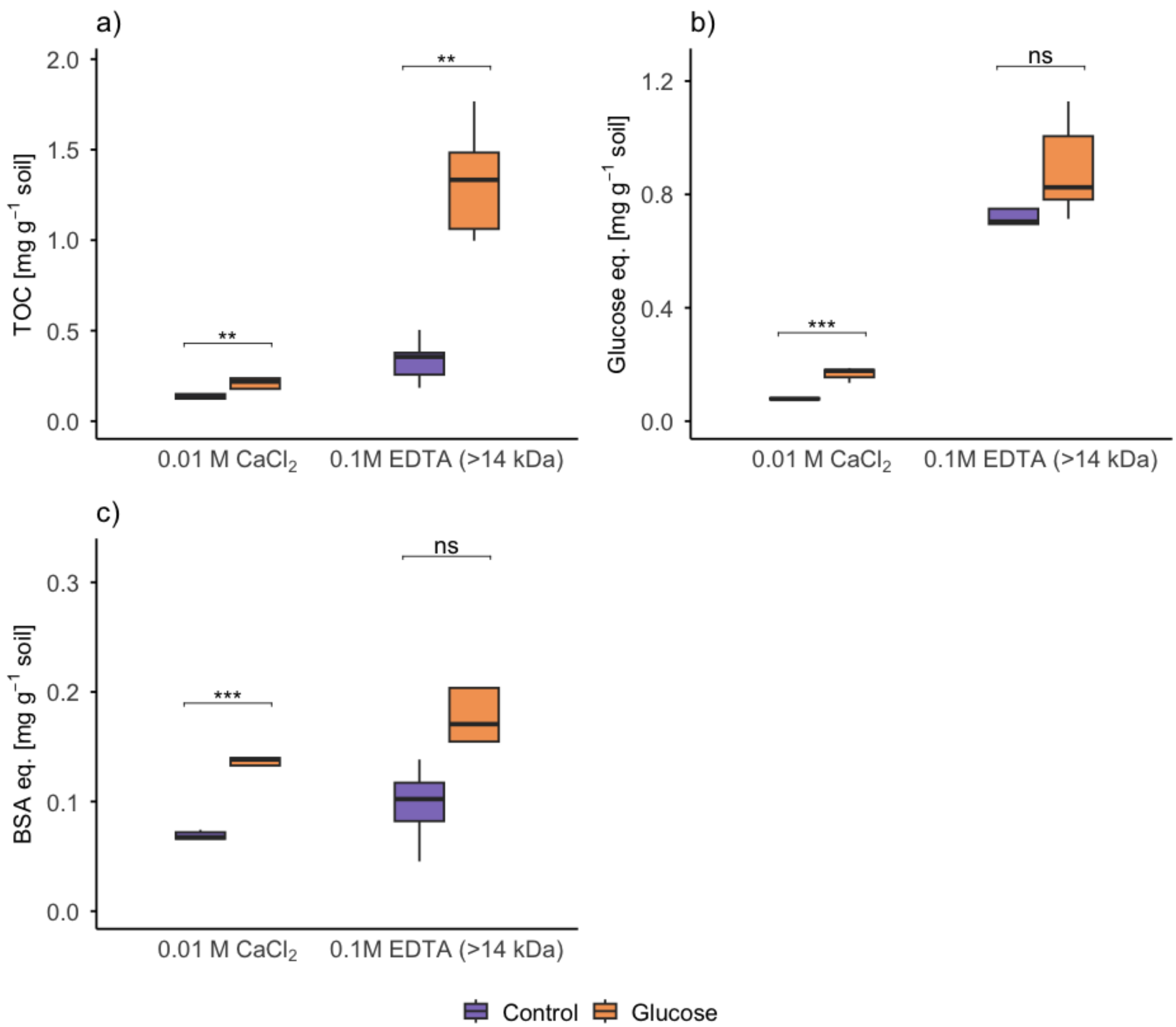

*Figure S5: Total organic carbon (a), polysaccharide (b) and protein (c) concentrations in 0.01M $CaCl_2$ and 0.1 M EDTA (>14 kDa) extracts of the control (deionized water) and the 2 wt.% glucose treatment. Asterisks indicate significant differences ($p < 0.01$ = **: $p < 0.001$ = ***).*

S10. Soil cation exchange capacity

To test the presence of soluble salts, both soils were mixed with deionized water at a 1:5 (w/v) ratio and shaken for 1h in an overhead-shaker. The electrical conductivity of the mixture was assessed using a Swan Chematest 35 (Swan, Hinwil, Switzerland). The low values for both soils (loam: 705 $\mu S\ cm^{-1}$, sandy loam: 534 $\mu S\ cm^{-1}$) indicated that washing steps for salt removal were not necessary. For cation exchange capacity (CEC) measurements, triplicates of soil were mixed with 1M NH_4Cl at a 1:5 ratio (w/v) in 50 mL tubes and shaken for 30 min. in an overhead shaker. Tubes were then centrifuged at

2500 *g* for 5 min. the supernatants collected. This step was iterated twice and the sample filtered through 0.45 μm polyethersulfone filters (Whatman, Maidstone, England). Measurements of acidified samples were conducted with an inductively coupled plasma Optical Emission Spectrometry (ICP-OES, 5100, Agilent Technologies, Basel, Switzerland) and analyzed for total dissolved Ca, Mg, Na, K, Mn, Fe and Al, usually representing the most abundant cationic elements in soils. CEC values of the soils as listed in Table S6 correspond to the sum of element charges obtained by multiplying the measured element concentrations with their respective valencies.

Table S6: Exchangeable cations and CEC of the loam and the loamy sand determined by 1M NH_4Cl extraction.

Soil	Ca^{2+} $cmol_c\ kg^{-1}$	Mg^{2+} $cmol_c\ kg^{-1}$	Na^{+} $cmol_c\ kg^{-1}$	K^{+} $cmol_c\ kg^{-1}$	Mn^{2+} $cmol_c\ kg^{-1}$	Fe^{2+} $cmol_c\ kg^{-1}$	Al^{3+} $cmol_c\ kg^{-1}$	CEC $cmol_c\ kg^{-1}$
Loam	22.4 ± 0.6	1.6 ± 0.007	0.3 ± 0.02	0.5 ± 0.0004	<0.1	<0.1	<0.1	24.8±0.6
Loamy sand	19.3 ± 0.6	1.1 ± 0.03	0.3 ± 0.02	0.3 ± 0.01	<0.1	<0.1	<0.1	21.1±0.6

References

(1) Brunauer, S.; Emmett, P. H.; Teller, E. Adsorption of Gases in Multimolecular Layers. *J. Am. Chem. Soc.* **1938**, *60* (2), 309–319. https://doi.org/10.1021/ja01269a023.

(2) Kinniburgh, D. G.; Milne, C. J.; Venema, P. Design and Construction of a Personal-Computer-Based Automatic Titrator. *Soil Sci. Soc. Am. J.* **1995**, *59* (2), 417–422. https://doi.org/10.2136/sssaj1995.03615995005900020021x.

(3) Keizer, M. G.; van Riemsdijk, W. H. ECOSAT, 2006.

(4) Bérard, A.; Clavel, T.; Le Bourvellec, C.; Davoine, A.; Le Gall, S.; Doussan, C.; Bureau, S. Exopolysaccharides in the Rhizosphere: A Comparative Study of Extraction Methods. Application to Their Quantification in Mediterranean Soils. *Soil Biol. Biochem.* **2020**, *149*, 107961. https://doi.org/10.1016/j.soilbio.2020.107961.

(5) Frølund, B.; Griebe, T.; Nielsen, P. H. Enzymatic Activity in the Activated-Sludge Floc Matrix. *Appl. Microbiol. Biotechnol.* **1995**, *43* (4), 755–761. https://doi.org/10.1007/BF00164784.

(6) Redmile-Gordon, M. A.; Armenise, E.; White, R. P.; Hirsch, P. R.; Goulding, K. W. T. A Comparison of Two Colorimetric Assays, Based upon Lowry and Bradford Techniques, to Estimate Total Protein in Soil Extracts. *Soil Biol. Biochem.* **2013**, *67*, 166–173. https://doi.org/10.1016/j.soilbio.2013.08.017.

(7) DuBois, Michel.; Gilles, K. A.; Hamilton, J. K.; Rebers, P. A.; Smith, Fred. Colorimetric Method for Determination of Sugars and Related Substances. *Anal. Chem.* **1956**, *28* (3), 350–356. https://doi.org/10.1021/ac60111a017.
